# Label-free quantification of imaging features in the extracellular matrix of left and right-sided colon cancer tissues

**DOI:** 10.1038/s41598-024-58231-3

**Published:** 2024-03-29

**Authors:** B. Arora, A. Kulkarni, M. A. Markus, P. Ströbel, H. Bohnenberger, F. Alves, F. Ramos-Gomes

**Affiliations:** 1 Translational Molecular Imaging, Max-Planck-Institute for Multidisciplinary Sciences, Hermann Rein-Straße 3, 37075 Göttingen, Germany; 2 Institute of Pathology, University Medical Center Göttingen, Robert-Koch-Straβe 40, 37075 Göttingen, Germany; 3 Clinic for Haematology and Medical Oncology, Institute of Interventional and Diagnostic Radiology, University Medical Center Göttingen, Robert-Koch-Straβe 40, 37075 Göttingen, Germany; 4 Cluster of Excellence “Multiscale Bioimaging: from Molecular Machines to Networks of Excitable Cells” (MBExC), University of Goettingen, Göttingen, Germany

**Keywords:** Cancer imaging, Cancer microenvironment, Gastrointestinal cancer

## Abstract

The molecular pathogenesis of colorectal cancer is known to differ between the right and left side of the colon. Several previous studies have focussed on the differences in clinicopathological features, proteomic and genetic biomarkers, the composition of gut microbiota, response to therapy, and the characteristics of the tumour microenvironment. However, the morphology and density of collagen in the extracellular matrix (ECM) have not been studied intensively. In this study, we employed 2-photon laser scanning microscopy (2PLSM) to visualise the intrinsic second-harmonic generation (SHG) signal emitted by collagen fibres in the heterogeneous ECM of human colon tumour tissues. Through texture analysis of the SHG signal, we quantitatively distinguished the imaging features generated by structural differences of collagen fibres in healthy colon and cancers and found marked differences. The fibres inside of tumours exhibited a loss of organisation, particularly pronounced in right-sided colon cancer (RSCC), where the chaotic regions were significantly increased. In addition, a higher collagen content was found in left-sided colon cancer (LSCC). In future, this might aid in subclassification and therapeutic decisions or even in designing new therapy regimens by taking into account the differences between collagen fibres features between colon tumours located at different sides.

## Introduction

**Figure 1:**
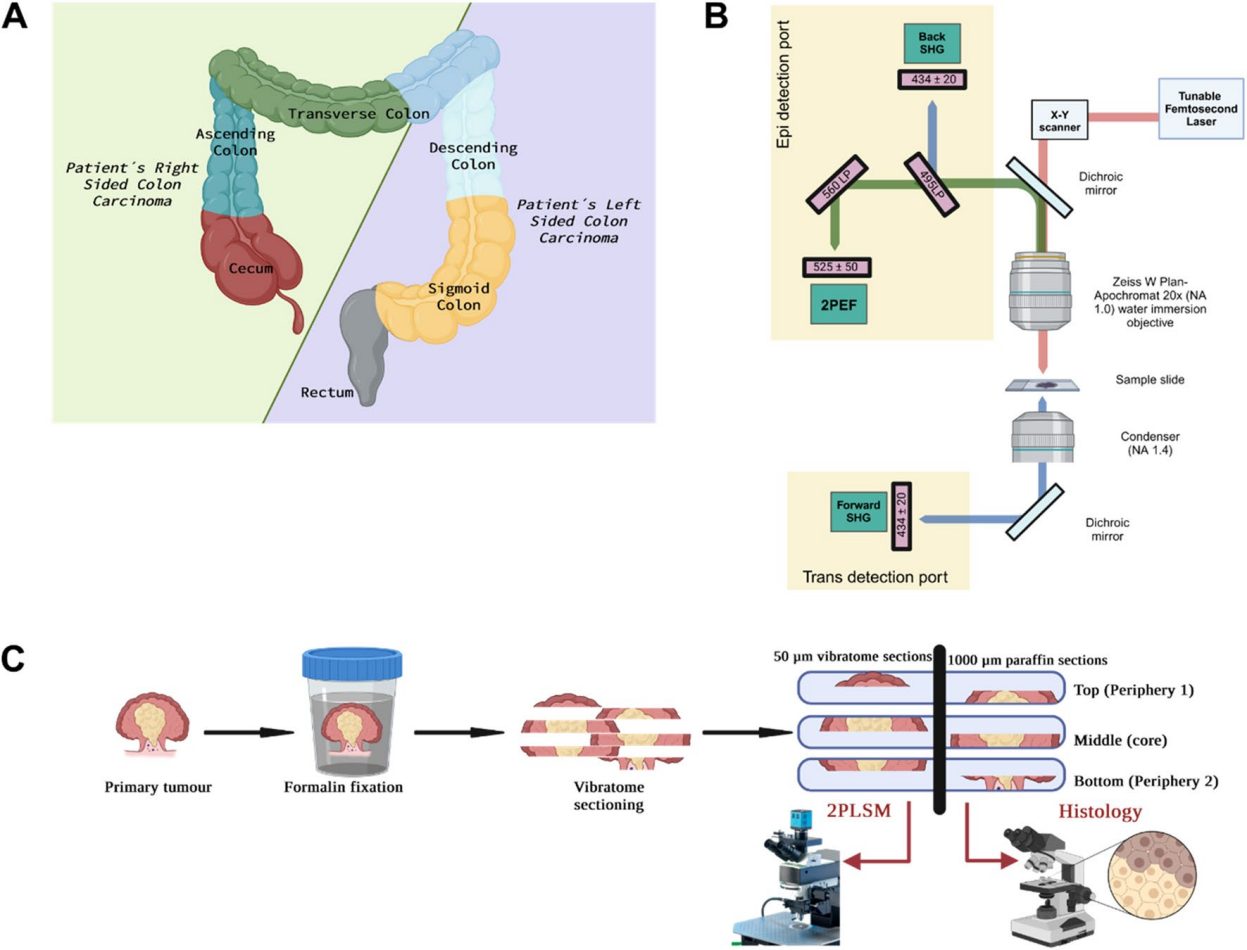
Sample origin and processing workflow. (**A**) Schematic presentation of the sites from which LSCC and RSCC samples were obtained. (**B**) Schematic of the optical setup. The excitation wavelength of 870 nm illuminates the tissue and the photons are scattered in the forward and back direction. The condenser collects the forward scattered photons and the light passes through a 434/20 nm filter to collect F-SHG signal by the PMT. The light path of the backscattered photons is further split by 495 nm and 525 nm long pass filters. The 2PEF and B-SHG are collected at the PMTs after light passes through 525/50 nm and 434/20 nm bandpass filters, respectively. (**C**) Pre-processing of samples, as well as, sectioning and imaging protocol. Created with BioRender.com.

Colorectal cancer (CRC) is the second leading cause of cancer mortality worldwide, with a persistently poor prognosis. Various factors, including the “site of origin”, significantly impact the molecular pathology and prognosis of CRC^1–3^. Tumours originating on the distal side of the splenic flexure, i.e., part of the transverse colon, descending colon, sigmoid colon and rectum, are termed left-sided colon cancer (LSCC), while tumours originating proximal to the splenic flexure, i.e., caecum, ascending colon and transverse colon, are referred to as right-sided colon cancer (RSCC) (Fig. 1A)^4^.

The constantly evolving cancer genome generates intricate heterogeneity not only in different kinds of tumours but also within each tumour itself. This dynamic nature of the tumour genome and proteome further reinforced the notion that they vary depending on the anatomical location^5–7^. Numerous studies have reported profound disparities in the proteogenomic, immunological landscape, and microbiota in LSCC versus RSCC^8–10^. Furthermore, these studies have revealed a perplexing variability in patient response to different therapies based on tumour location^11–13^.

The role of the tumour microenvironment and its extracellular matrix (ECM) in influencing the pathophysiology of cancer has gained traction in the past few years. Collagen, a major ECM component, has garnered particular attention due to its paradoxical and multifaceted influence on cancer progression. It acts as a double-edged sword, which can help in promoting tumour progression by increasing metastasis or stopping tumour cells from infiltrating other regions.^14,15^. However, the ECM and collagen fibre organisation is notoriously hard to describe. The routinely used histochemical staining for collagen including the three-colour Masson’s trichrome staining^16^, and Picrosirius staining^17^ are not very precise in determining fibres features. Moreover, the staining results are not easily quantifiable. To stain collagen subtypes, specific antibodies are more useful, but even in such a case, it is challenging to obtain additional structural features. The histological analysis is subjective and qualitative rather than quantitative^18^. Leveraging the structural properties of collagen fibres, 2-photon laser scanning microscopy (2PLSM) has emerged as a promising imaging tool to investigate the role of collagen in tumorigenesis^19,20^. The non-centrosymmetric collagen fibre structure generates an intrinsic second-harmonic generation (SHG) signal, allowing for specific imaging without extensive sample preparation or staining procedures^21^. Specifically, the fibrillar collagen subtypes (collagen type I, II, III, V, XI, XXIV, XXVII) generate the SHG signal in the cancer stroma^22^. It is noteworthy that the SHG signal has high selectivity, sensitivity and submicron-level resolution for collagen fibres, which cannot be reached by conventional histological staining methods^23^. The 2-photon excitation fluorescence (2PEF) helps to visualize the tissue morphology without any labels due to the autofluorescence emitted by cells and components of ECM of tissues^24^. Research is being done to understand how the SHG signal might alter depending on the sample preparation methods so that its effect can also be taken into consideration while performing 2PLSM studies^25^.

Our 2PLSM imaging workflow identified key imaging features from the SHG signal generated by collagen fibres present in human colorectal tissues. Texture analysis on the collagen fibres allowed quantification and accurate assessment of collagen amount, degree of collagen fibre organisation and the extent of collagen fibre waviness in CRC tissues and thereby accurate differentiation between healthy and tumorous tissues. These potential label free imaging biomarkers showed differences in CRC tumours based on the anatomical location, distinguishing between LSCC and RSCC by the higher collagen fibre content found in LSCC. Interestingly, RSCC exhibited also a lower degree of collagen fibre organisation in comparison to LSCC.

## Material and methods

### Tumour sample collection

The study cohort consists of human tumour tissues resected from patients with RSCC or LSCC (n = 17) (Fig. 1A). Both the LSCC and RSCC cohort have a similar distribution of tumour stages and morphology as detailed in the sample information table in the supplementary file (Table 1: sample information). From ten of these patients, healthy colon tissues were also obtained during surgery. The human tissue samples were collected and used according to the approval and consent of the Ethics Committee of the University Medical Center Goettingen (Ethics approval #5/10/17). All patients provided written informed consent for the use of pathology specimens for research purposes. The study was performed in accordance with the Declaration of Helsinki.

### Sample preparation

Tumour tissues as well as healthy controls were obtained fresh or formalin-fixed. If the sample was fresh, they were first fixed in 4% paraformaldehyde solution (PFA) overnight at room temperature (RT). Figure 1C shows a schematic workflow of the sectioning procedure. The tumours were embedded in 5% agarose and were cut into 50 µm and adjacent 1 mm sections using a vibratome (VT1000 S; Leica Biosystems). The 50 µm tissue sections were mounted on glass slides with Aquapolymount mounting medium before imaging by 2PLSM. The 1 mm sections were embedded in paraffin for histology and immunohistochemical staining.

### Label-free imaging

Label-free SHG and 2PEF images were acquired using an upright TriM Scope II multiphoton microscope (Miltenyi Biotec, Bielefeld, Germany) equipped with a tunable femtosecond laser with two independently tunable output channels (Cronus 2P, Light Conversion, Vilnius, Lithuania). The first tunable channel (680–960 nm) was used for SHG and 2PEF excitation. The laser operated at 15% of its maximal output power (1.0 W). The incident laser power under the objective was 19 mW at 870 nm. Images were acquired using a Zeiss W Plan-Apochromat 20× (NA 1.0) water immersion objective. The backscattered emitted light was split by 495 nm and 560 nm long pass dichroic mirrors (Semrock) and detected through the objective lens at a photomultiplier tube (PMT) and GaAsP detectors (Hamamatsu). A PMT in the transmission position collected the forwardscattered emitted light gathered by a 1.4 NA condenser lens under the stage. SHG, and 2PEF signals were collected using the filter settings 434 ± 20 nm and 525 ± 50 nm, respectively (BrightLine HD filters, Semrock, AHF Analysentechnik Tübingen, Germany). Collagen fibre signals were collected as either backscattered SHG (B-SHG) or forwardscattered SHG (F-SHG). The 2PEF signal was collected as backscattered. The optical setup depicts (Fig. 1B) the excitation and emission pathways for 2PEF, B-SHG and F-SHG. 2D mosaics of entire sections were acquired with individual image sizes of 199 × 199 µm with 512 × 512 pixels, a pixel dwell time of 3.79 µs with two-fold averaging and 10% overlap within each tile of the mosaic.

### Histology and immunohistochemistry

The formalin-fixed, paraffin-embedded tissue sections of 1 mm thickness taken from regions adjacent to tissue sections used for 2PLSM were cut into 2 µm slices and mounted on glass slides (Fig. 1C). The tissue sections were deparaffinised and Haematoxylin & Eosin (H&E) and Masson’s Trichrome staining (MTS) were performed as previously described^26,27^.

Tumour cells in tissues were stained by performing immunohistochemistry with an antibody against the human epidermal growth factor receptor (EGFR), as previously described^28^. Briefly, slices were incubated overnight with rabbit-anti-human EGFR antibody (Thermo Fisher Scientific, clone SP9; 1:500) followed by 1 h incubation with N-Histofine Simple Stain Max PO anti-rabbit (Nichirei) and stained with 3-amino-9-ethylcarbazole (AEC). Images were acquired with an Axiovert 200 M inverted microscope (Carl Zeiss Microscopy GmbH). Image generation and processing were performed with Axiovision Rel.4.6 and Fiji, respectively.

### Image processing

**Figure 2:**
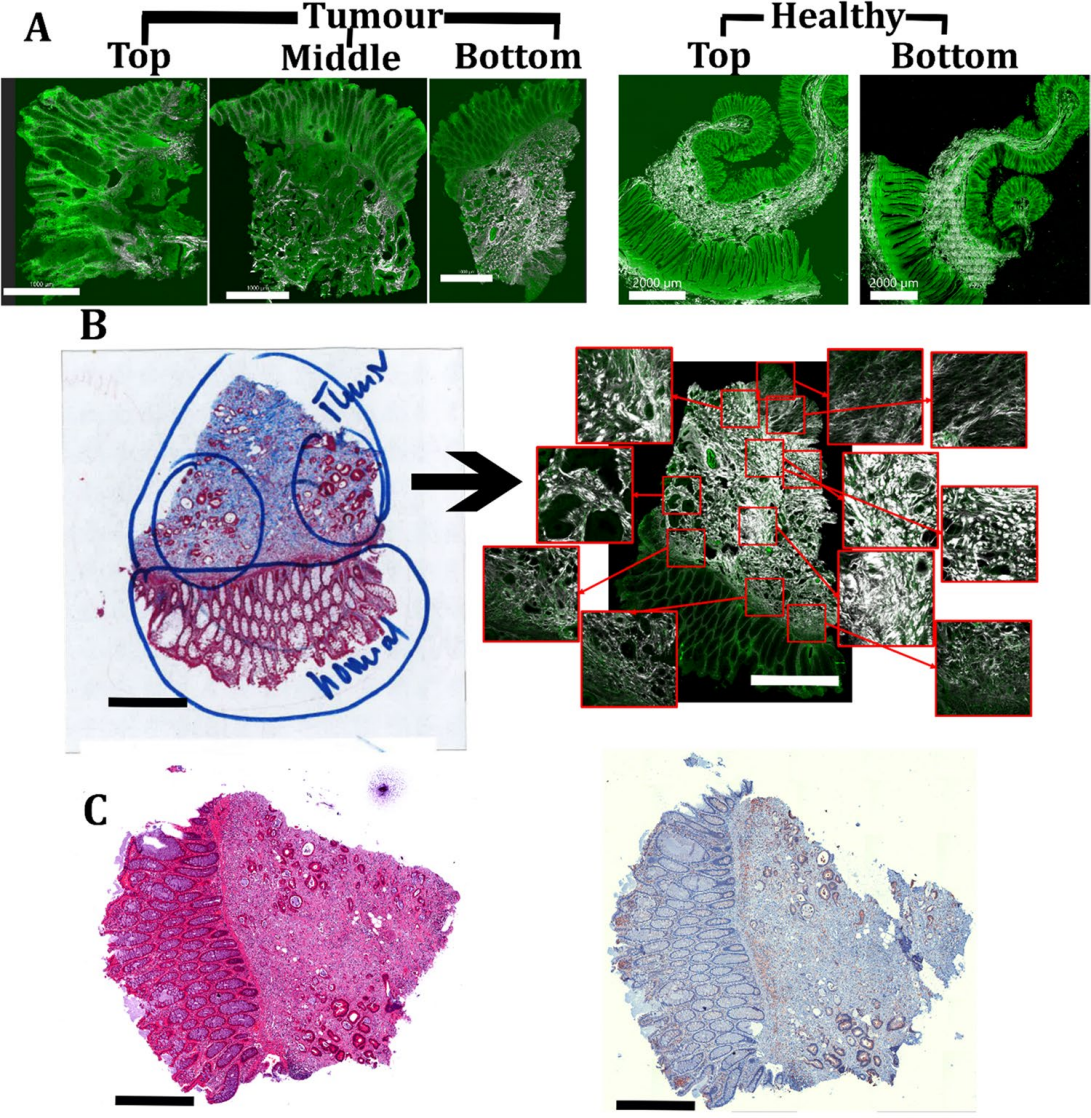
Addressing tumour heterogeneity and selecting tumour ROIs. (**A**) Higher intra-tumour heterogeneity in colon tumour tissues compared to healthy colon tissues (Vibratome sections from top, middle and bottom of a LSCC; top and bottom sections of healthy sample), as seen in the 2PLSM image. (**B**) Selection of ROIs by a pathologist in a representative MTS stained bottom section (left) and selection of 10 ROIs/section in a 2PLSM mosaic image (right). The zoomed in ROIs are highlighted in the insets. White illustrates F-SHG signal and green autofluorescence signal. (**C**) Assessment of representative colon tumour paraffin sections by H&E (left panel) staining and immunohistochemistry using an antibody against EGFR (right panel) which is highly expressed in the tumour cells. Scale bar represents in (**A**): 1000 μm in tumour tissue and 2000 μm in healthy tissue, (**B**) and (**C**): 1000 μm.

The 2PLSM mosaic images were used for selecting regions of interest (ROIs) of 394 × 394 µm^2^. A total of 10 ROIs/section were selected for each 3 slices (top, middle and bottom) of tumour and 10 ROIs/section for each 2 slices (top and bottom) of healthy tissue. (Fig. 2B) i.e., 30 ROIs/tumour and 20 ROIs/healthy tissue were scanned/ imaged for quantification. Each image was split into F-SHG, B-SHG and 2PEF channels. The 2PEF channel was used to visualise the tissue morphology. Since the SHG signal from the collagen fibres observed in the F-SHG channel provided a higher pixel resolution compared to the B-SHG channel, only the F-SHG signal was used for quantification. The F-SHG image was denoised using global Otsu adaptive thresholding. To calculate the percentage area occupied by the collagen fibres in each ROI, the sum of the pixel intensities over the threshold value was calculated for the F-SHG channel. This was performed in a batch process using an in-house macro, written in FIJI. The images were then converted to 8-bit tiffs using FIJI for further processing.

Furthermore, to investigate the morphology and the spatial organization of the collagen fibres, texture analysis was performed. Two commonly used scalar parameters, namely orientation and coherence of collagen fibres were quantified. The local orientation and the coherence were obtained through a structure tensor-based methodology, where the structure tensor is a 2-by-2 matrix constructed around each pixel in the image for a given σ, defined as the local area of interest around each pixel in the image^29^. Once the structure tensor was formulated, the local orientation (bounded between 0 and 180 degrees with respect to the x axis) was calculated as the direction of the dominant eigenvector of the structure tensor. This provided information about the local orientation of the collagen fibres within the image. Additionally, the coherence was defined as the ratio between the differences and the sum of the structure tensor eigenvalues^29^. Coherence is thus bounded between 0 and 1, where 1 indicates highly aligned structures and 0 indicates disorder. This measure allowed for the spatial characterization of the overall organization and alignment of the collagen fibres in the image.

### Statistics and reproducibility

For statistical analysis, unpaired, two-tailed t-test was performed to compare healthy with the tumour group. An ordinary one-way ANOVA was performed to compare the four subgroups, i.e., LSCC, RSCC, healthy-left and healthy-right. The analysis was performed using Graph Pad Prism 9 with a p-value of 0.05 as a margin for statistical significance. Data is presented as mean ± SD, * indicates p ≤ 0.05, ** indicates p ≤ 0.01, *** indicates p ≤ 0.001, **** indicates p ≤ 0.0001.

## Results

### Development of a comprehensive sectioning-imaging protocol to account for intra-tumour heterogeneity

Since the organisation of individual tumours and their microenvironment is inherently heterogenous (intra-tumour heterogeneity), we developed a comprehensive sectioning protocol tailored to encompass a wide array of their features. We divided each tumour sample into top, middle and bottom sections (Fig. 1C). Some tumour samples were very small, for which only top and bottom sections were performed. Notably, the remarkable intra-tumour heterogeneity in collagen fibre distribution, as revealed by 2PLSM images, is illustrated in Fig. 2A. A clear contrast can be observed when comparing the heterogeneity in top and middle and bottom sections of a tumour with the homogeneity from healthy colon, top and bottom sections (Fig. 2A). Here, the F-SHG signal emitted by collagen fibres is represented in white, and the 2PEF signal emitted by the cells and ECM of the colorectal tissue is shown in green.

Since healthy colon tissue presents a high homogeneity throughout the sample, we only used two sections (top and bottom) for the analysis. The morphological architecture of collagen fibres in the healthy sections demonstrate a remarkable similarity between the top and bottom section (Fig. 2A, right). Distinct boundaries can be observed within the various layers of the colon, presenting a uniform structure comprising of crypts, submucosa, and muscular and serosa layers. The submucosa and serosa regions exhibit thick bundles of wavy fibres. The collagen fibres form circular pattern around the crypts (Fig. 2A, right). In contrast, the tumour sections exhibit a disorganised mesh of both straight and wavy fibres, lacking clear organisation within and between the layers of the colon. Although the tumour border is demarcated from the healthy colon in the bottom section, it becomes challenging to delineate it in the top and middle section. This example of LSCC tissue has a higher prevalence of straight fibres in the middle section (Fig. 2A, left).

These findings emphasise the necessity of obtaining multiple sections from each tumour sample to capture a wide variety of morphological features present in one tumour. This aids in a comprehensive analysis of tumour tissues.

Accurately differentiating between the healthy and neoplastic regions and distinguishing the tumour from other tissues such as muscle and blood presents an intricate challenge. To address this problem, the pathologist marked the tumour ROI in the tissues’ adjacent MTS stained histological images (Fig. 2B, left panel). Subsequently, we extracted the ROIs from the corresponding regions in the adjacent 2PLSM section. The ROIs are shown as zoomed-in images in insets (Fig. 2B, right panel). MTS staining facilitated the comparison of the F-SHG signal from 2PLSM to the connective tissue, specifically collagen fibres. The H&E staining helped in validating the tumour (Fig. 2C, left panel). Furthermore, the positive staining of tumour cells by the antibody targeting EGFR, highly expressed in colon cancer cells, validated the presence and location of tumour cells in the tissue (Fig. 2C, right panel).

We additionally analysed tissue samples from the healthy regions next to the tumour tissues for comparison (Fig. 2A, right). ROIs were obtained from all layers of the healthy colon tissue to ensure the inclusion of the morphological diversity of collagen fibres from all the physiological regions (such as crypts, submucosa, muscularis and serosa). Through the integration of histological evaluation in conjunction with 2PLSM imaging, we ensured that the selected ROIs captured the relevant healthy and colon cancer tissue areas for further analysis and quantification.

### Anatomical origin influences tumour ECM variations

**Figure 3:**
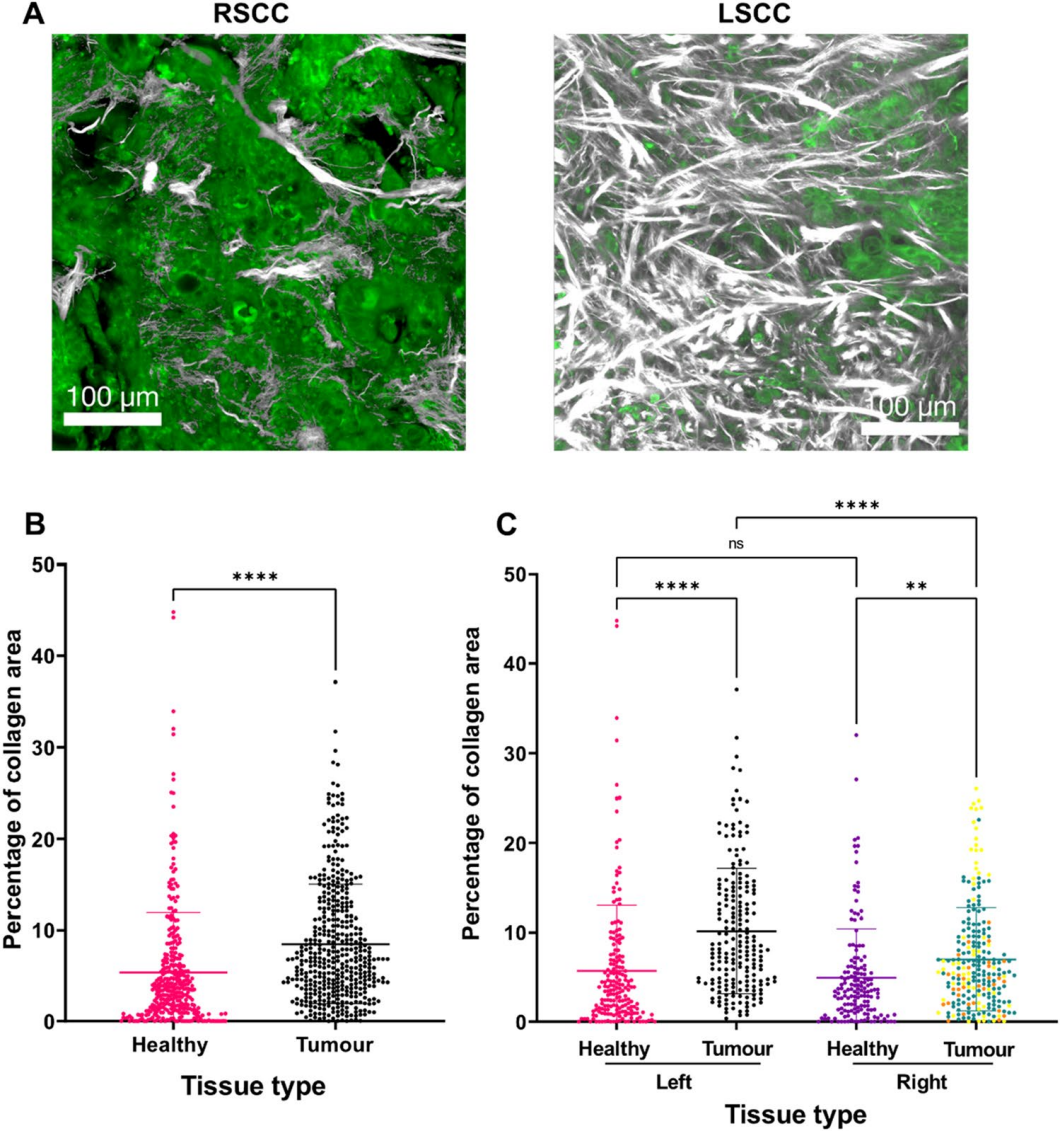
Collagen fibre content in LSCC v/s RSCC and healthy colorectal tissues. (**A**) Representative images of 2PLSM ROIs of the middle tumour sections obtained from RSCC (left) and LSCC (right). White is F-SHG signal and green is 2PEF signal. Scale bar in A represents 100 μm. (**B**) Higher percentage of the occupied collagen fibre area in ROIs of each group indicates that tumour tissues (n = 17) have higher collagen fibre areas than healthy tissues (n = 10). (**C**) Amount of collagen fibres in LSCC (n = 8) is higher than in RSCC (n = 9). Orange points indicate ROIs from a CRC derived metastasis and yellow points indicate ROIs from microsatellite instability (MSI) colon cancer samples. No significant difference was found in healthy left (n = 6) compared to healthy right (n = 4) colon tissue. Scale bar represents in (**A**): 100 μm. Data is presented as mean ± SD, ** indicates p ≤ 0.01, *** indicates p ≤ 0.001, **** indicates p ≤ 0.0001.

Quantification of the F-SHG signal in both healthy and tumour regions from LSCC and RSCC revealed significant differences in collagen fibre content. In Fig. 3A, we show representative images of ROIs obtained by 2PLSM highlighting the contrast of the ECM features between the middle sections of an LSCC and an RSCC tumour. Our findings revealed a significant increase in the percentage area occupied by collagen fibres in tumour tissues ROIs compared to healthy tissue ROIs (Fig. 3B; p < 0.0001). Furthermore, we report a marked distinction in the amount of collagen fibres between tumours originating from LSCC and those originating from RSCC, with the former displaying a significantly higher collagen fibre occupancy (Fig. 3C; p < 0.0001). Although the RSCC tumour sample group contains a CRC derived metastasis and two colon tumours with microsatellite instability (MSI) in contrast to the LSCC group (Suppl. Table 1), these variants did not impact the statistically significant difference in collagen area between LSCC and RSCC (data not shown). Additionally, the RSCC and LSCC tumour groups include two and one G3 stage tumours, respectively (Suppl. Table 1 and highlighted in Suppl. Fig. 1), which also did not have an effect in the collagen content differences between left and right tumours (data not shown). These findings emphasise the need for considering the tumour ECM when studying the CRC tumour microenvironment since amount and morphology of collagen fibres can vary not only within the individual tumour but are also dependent on the anatomical side, where the colon tumour developed.

### Texture analysis of collagen fibres introduces quantifiable biomarkers

**Figure 4:**
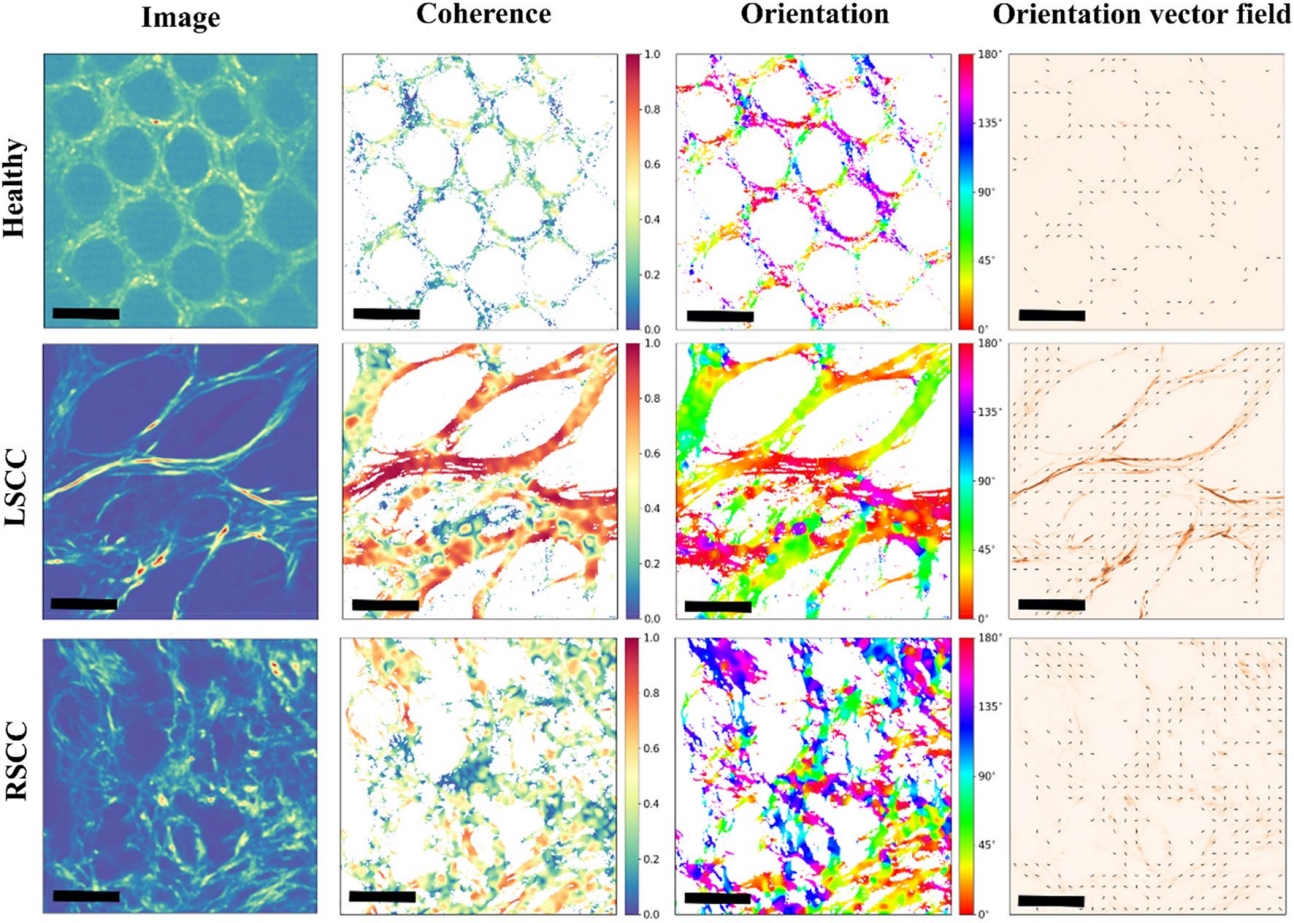
Texture analysis of collagen fibres. Representative images of 2PLSM ROIs are shown along with the coherence map, orientation map and orientation vector field of healthy (upper row), LSCC (middle row) and RSCC (lower row) middle tissue sections. The coherence values range from 0 (blue) indicating chaotic mesh of collagen fibres to 1 (red) highlighting organised and aligned fibres. The orientation values range from 0 to 180 degrees representing the angle of collagen fibres. The orientation vector field displays the dominant direction of the F-SHG signal. Note, that the coherent regions with a value of 1 are highest in the healthy tissues, followed by LSCC and then RSCC. Scale bar represents 100 μm.

To quantify the morphology of collagen fibres in the tumour ECM, we conducted texture analysis on the F-SHG signal within each ROI. Orientation and coherence maps were generated by determining the dominant direction of pixel intensity in each local window of interest. For statistics, the mean values of coherence and orientation were calculated for each ROI. The accuracy of this approach is substantiated by the precise masks generated in the orientation maps (Fig. 4). This mask replicates the morphology of collagen fibres in each image precisely.

The local coherence map illustrates the organisation of collagen fibres, with values ranging from 0 (indicating chaos) to 1 (representing highly aligned fibres), regardless of their straight or wavy nature. The local orientation indicated the direction of the collagen fibres represented by circular values ranging from 0 (blue) to 180 degrees (red). These maps served as valuable tools to quantify the structural characteristics of collagen fibres. The coherent regions with a value of 1 are highest in the healthy tissues, followed by LSCC and then RSCC.

### Quantification of collagen fibre waviness and coherence provides valuable insights into tumour morphology

**Figure 5:**
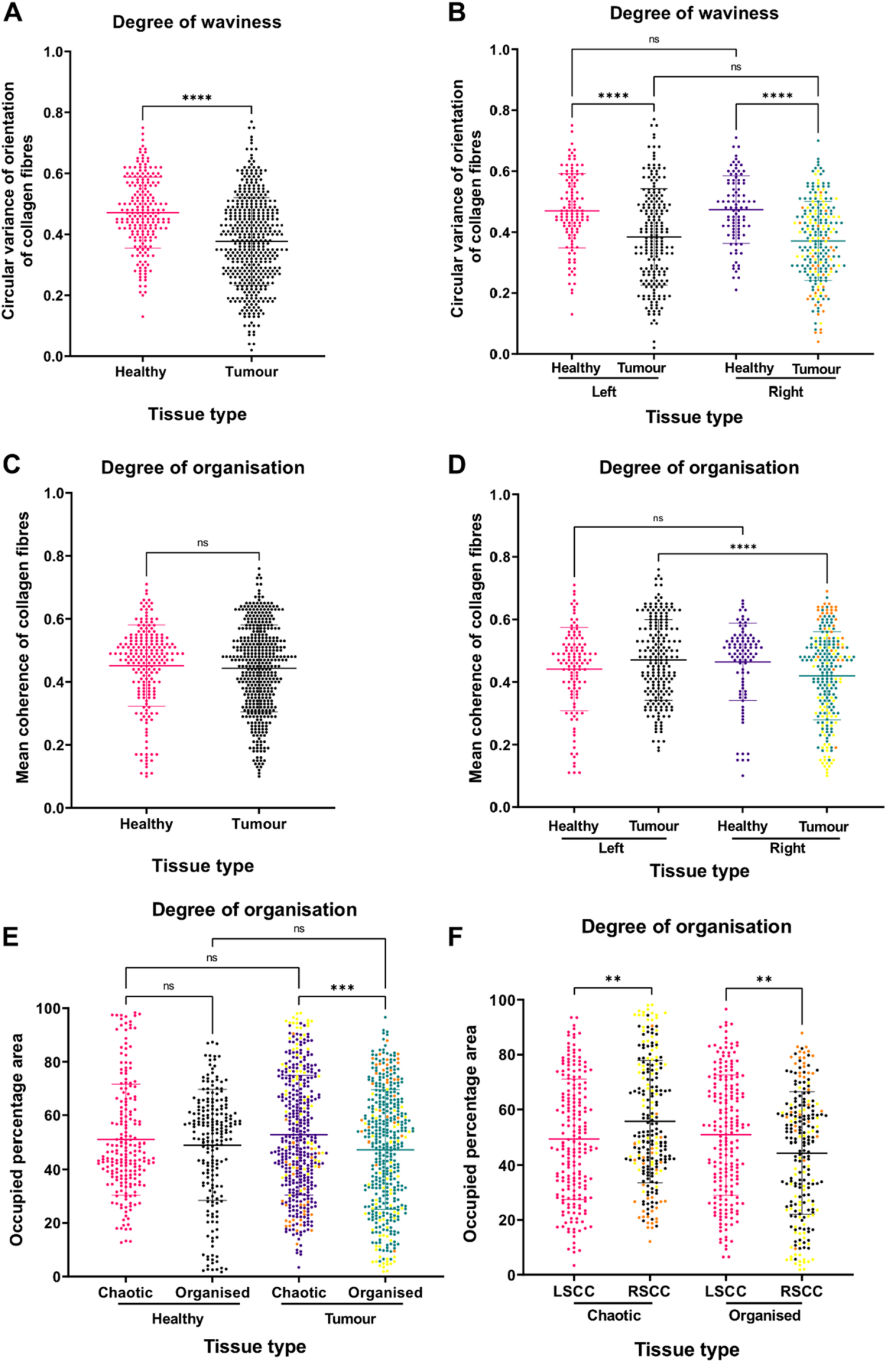
Differences in the imaging features of collagen fibres obtained by texture analysis on the F-SHG signal from 2PLSM images. (**A**) Collagen fibres in tumour regions are straighter compared to the healthy tissues. (**B**) The waviness of the fibres does not change in left and right sided colon cancer. (**C**) Coherence cannot segregate the tumour from healthy tissues. (**D**) Coherence can distinguish the LSCC, which has a higher degree of organisation than the RSCC. (**E**) The percentage of regions of low organisations in tumours increases significantly whereas healthy tissues have uniform distribution of organised and unorganised structures. (**F**) The low coherence (chaotic) regions in RSCC are higher than LSCC. Orange points indicate ROIs from a CRC derived metastasis and yellow points indicate ROIs from MSI tumour samples. Data is presented as mean ± SD, ** indicates p ≤ 0.01, *** indicates p ≤ 0.001, **** indicates p ≤ 0.0001; ns = not significant.

We sought to quantify the degree of waviness of collagen fibres by calculating the circular variance of their local orientation. A higher variation in orientation angles indicates higher fibre waviness. Our analysis revealed that the healthy tissues predominantly consist of collagen fibres, that are densely present in the submucosa region and are arranged in a circular pattern around the crypts. In contrast, tumour tissues exhibit relatively straight collagen fibres without significant variations based on the anatomical origin (Fig. 5A, B; p < 0.0001). Hence, waviness alone was a reliable feature of tumour v/s healthy collagen fibres, however, this parameter did not facilitate differentiation between the anatomical origins of colon cancer.

Therefore, we explored the mean coherence as an imaging feature to distinguish between the LSCC and RSCC tumours. While mean coherence was not able to separate healthy and tumour tissues (Fig. 5C), the RSCC tumours exhibited greater chaos than the LSCC (Fig. 5D; p < 0.0001). To comprehend the differences in coherence, we sub-classified mean coherence features into low and high coherent regions based on the mean coherence values of (0 to 0.5) and (0.5 to 1), respectively, for each sub-group. We then calculated the percentage area occupied by each type of fibres in the ROIs. We found that the healthy tissues have a homogenous distribution of organised and chaotic regions of collagen fibres. The crypts displayed organised regions, while the submucosa showcased a chaotic mesh primarily composed of wavy fibres (Fig. 5E). However, the highly chaotic regions in tumour tissues, particularly within the crypts, significantly increased (p = 0.0003). Furthermore, the percentage area occupied by these chaotic regions was higher in the tumours originating from the RSCC than the LSCC (p = 0.0096). These chaotic regions comprised a heterogenous mix of straight and wavy fibres (Fig. 5F). Similar to the parameter ‘collagen area’, the metastasis, the 2 MSI tumours in the RSCC group as well as the G3 stage tumours (Suppl. Table 1) did not impact the statistically significant difference that we found in all fibre parameters between LSCC and RSCC (data not shown).

## Discussion

This study introduces a comprehensive and standardised methodology for label free multiphoton imaging in spatially heterogeneous tissues such as tumours. Our study introduces an open-source tool to quantify the SHG imaging features of tumour morphology in a standardised manner. This was achieved by performing texture analysis on the collagen fibres of CRC as a proof of principle, which identified novel quantifiable morphological differences between LSCC and RSCC based on the distribution of F-SHG signal. While one feature alone may not segregate all the tissue subgroups, we demonstrate that their combination helps to characterize the ECM feature of a given tissue quantitatively and might provide valuable insights into the ECM organisation of colon cancer. We focused on collagen fibre amount, degree of collagen fibre disorganisation and the extent of collagen fibre waviness.

Our findings that the straightness of collagen fibres in tumour is higher than in healthy tissues, and thereby successfully distinguishes between healthy and tumour tissues, support the recent observation by Despotović et.al. stating that cancerous tissues exhibit relatively straight fibres compared to healthy colorectal tissues^30^. Our observation further supports the notion that the invasion capability of cancer tissues could be facilitated by the ability of ECM, specifically collagen fibres, to change their morphology, straighten, and form streaks of collagen paths, all enabling cancer cells to migrate to surrounding tissues^14^.

Over the past decade, significant advancements have been made in the applications of 2PLSM to differentiate healthy ECM from tumour ECM. In our study, we identified differences not only in the waviness of collagen fibres, but also demonstrate that the ECM of tumour tissues has a higher collagen deposition than that of healthy tissues. The latter finding is in line with a previous study by Birl et al.^31^ showing, that the average intensity per pixel of collagen in the ECM of malignant tumours is significantly higher than the normal colonic mucosa, low grade intraepithelial neoplasia (LGIP) and high grade intraepithelial neoplasia (HGIP). In addition, it was reported that the density of collagen in the basement membrane (BM) reduces from normal over precancerous to cancerous colonic tissues based on the quantification of the SHG signal in the BM^32^. Similarly, pixel density of collagen has been shown to reduce from normal, over LGIP to HGIP in the mucosa layer^33^. Similar findings were reported in the muscularis layer, where the number of collagen pixels in normal tissue was shown to be higher than in carcinogenic tissues^34^. In contrast to these studies, our work takes into consideration all the anatomical layers when comparing the healthy to tumour ECM. Thus, the conclusions drawn in our study are not subjective to or limited by the anatomical layer in which tumour is present, since we have taken ROIs from all the healthy tissue layers, including the crypts, submucosa and serosa.

By combining label free 2PLSM imaging, ROI identification and texture analysis, we show that the collagen phenotype, mainly structural features of collagen fibres, differs between LSCC and RSCC tissues. We found that the ECM of RSCC tissues loses the degree of organisation to a higher order than that of LSCC. The organisation of collagen fibres in tumours can influence their metastatic and chemo-resistant capabilities. Using 2PLSM, Chiang, HC. et al. have already shown that the alignment of collagen fibres is critical in conferring resistance against immune cells^35^. In-vivo and in-vitro studies have proven that a mesh-like network of Collagen type IV (COLIV) assists in providing chemoresistance to lung and breast tumours^36^. Such studies underline that measuring coherence or degree of organisation might aid a better understanding of cancer pathophysiology. Our tumour sample cohort contained several variants, including two tumours with microsatellite instability (MSI), a unique molecular alteration and hyper-mutable phenotype that occurs in 15% of colorectal cancers^37^, a metastasis from colon adenocarcinoma (all in RSCC), as well as three stage G3 colon adenocarcinomas (two in RSCC and one in LSCC). Notably, none of these variants had an impact on the collagen differences between LSCC and RSCC, indicating that these variations do not influence the collagen phenotype.

By quantifying SHG signal from collagen fibres, we demonstrate that the amount of collagen fibres in tumours originating from LSCC is higher than RSCC. This finding aligns with the fact that the genes for expression of cyclooxygenase-2 (COX-2), an enzyme that plays a role in the inflammatory response to cancer, is upregulated in LSCC compared to RSCC^38^ and the overexpression of COX-2 in the tumour stroma is associated with high deposition of collagen^39^.

There is an ongoing debate on the question which side of colorectal cancer has a poorer prognosis^40^. While several studies advocate for RSCC to have a less survival rate^41^, a number of studies support the notion that RSCC has, in fact, a better outcome compared to LSCC. Some studies have also reported no difference in response to treatment in LSCC v/s RSCC^42^. Given the critical role of the ECM in tumour dormancy, metastatic potential and resistance to therapy^43,44^, understanding the characteristics of a major component of ECM, i.e., collagen, becomes of utmost importance in this type of cancer. Multiple studies underline the correlation between a high expression of collagen and an improved prognosis. A high expression of the α2 chain of collagen I (COL1A2), the major fibrillar component of ECM, has been identified as a novel tumour suppressor, which leads to inhibition of proliferation, migration and invasion in CRC cell lines^45^. Furthermore, the deletion of COL1 in myofibroblasts accelerates cancer progression and tumour-derived collagen type III (COLIII) promotes tumour dormancy^46^. These studies suggest that adding the collagen imaging features introduced in our study as an additional parameter may be important when comparing the patient prognosis between LSCC and RSCC. Identifying specific collagen fibre phenotypes in the future, might help explain not only the differential treatment response and chemoresistance of LSCC and RSCC, but may also support intervention decisions.

With the availability of large 2PLSM datasets and the advancements in machine learning, such “collagen signatures” have already been shown to be associated with clinical outcome^47–49^. Some of the 2PLSM studies have also shown the potential of accurate tumour subclassification. Chen et al. compared the SHG morphological features, such as fibre area, density, and cross-link density of collagen fibres in gastrointestinal stromal tumours against gastric adenocarcinomas by combining 2PLSM with automated image classification^47^. Another group classified the patients with and without radiotherapy using SHG characteristics such as collagen amount and fibre alignment^50^.

Our current study focused on 2D images and did not implement polarized SHG, however both the evaluation of entire volumes and simultaneous use of polarization may increase the information on collagen fibres and should be considered in future studies. Due to the inherent anisotropy of collagen, the SHG signal intensity is strongly dependent on the polarization direction of the incident laser beam relative to the orientation of collagen fibres. Maximum SHG signal is typically obtained when the polarization direction of the incident light is parallel to the collagen fibre axis. This is because SHG efficiency is highest when the incident polarization direction matches the orientation of the nonlinear optical dipoles within the collagen structure^51^. Changes in the SHG signals in dependence of polarization and in dependence of fibre direction within entire tissue volumes may thus supply additional information on the structural organization within the CRC tissue by providing spatial distribution and more detailed orientation of collagen fibres.

In the future, the morphological quantification introduced in our study could be incorporated into real-time 2PLSM studies, where currently the morphological analysis is quite subjective. The development of flexible laser scanning 2-photon endoscopes offer the distinct advantage of enabling in-depth visualisation of native tissues, in real-time, without labelling or causing minimal harm to the tissue^52^. The feasibility of real-time 2PLSM to discriminate colorectal cancer lesions^53^ and in identifying surgical margins in rectal cancers have been already demonstrated^54^, albeit not in a quantitative manner. Thus, collagen phenotypes as we show here might be implemented in 2PLSM endoscopes.

Our method has broad application potential not only in cancer imaging^55^, but also in other diseases associated with changes in the ECM, such as chronic inflammation^56,57^.

In conclusion, by quantifying and comparing the morphological characteristics of collagen fibres in LSCC, RSCC, and healthy tissue samples using 2PLSM, we not only shed light on the spatial heterogeneity of the ECM in colorectal cancer, but also distinguish between LSCC and RSCC tumours based on collagen amount, degree of collagen fibre disorganisation in tissues and the extent of collagen fibre waviness. Further clinical studies, on a larger cohort, are necessary to validate the use of the demonstrated features as imaging biomarkers for CRC subclassification. These findings contribute to a better understanding of the tumour microenvironment and present additional features that may correlate to cancer progression, metastasis, and treatment response.

### Supplementary Information

Supplementary Information.

## Data Availability

The datasets used and/or analysed during the current study available from the corresponding author on reasonable request.

## Code availability

The source code for the texture analysis is available at https://github.com/ajinkya-kulkarni/PyTextureAnalysis.
