# Supplementary material for: Label-free quantification of imaging features in the extracellular matrix of left and right-sided colon cancer tissues
Source: Sci Rep. 2024 Mar 29;14:7510. doi: 10.1038/s41598-024-58231-3 (PMC10980747; doi:10.1038/s41598-024-58231-3)
Supplement: Supplementary file 1 — Supplementary Information. [file 41598_2024_58231_MOESM1_ESM.docx]

**Supplementary Information**

**Supplementary Table 1**

**Suppl. Table 1**: TNM classification of tumours included in the left-sided colon cancer (LSCC) and right-sided colon cancer (RSCC) groups

| **Sample number** | **TNM classification** | **Grade**  **(G2/G3)** | **MSS/MSI status** | **LSCC/RSCC** |
| --- | --- | --- | --- | --- |
| 1 | peritoneal metastasis from a colon adenocarcinoma |  | MSS | RSCC |
| 2 | pT3 N2a (6/47) L1 V0 | G3 | MSS | LSCC |
| 3 | pT3 N1a (1/38) L1 V0 Pn0 | G2 | MSS | RSCC |
| 4 | pT3 N0 (0/15) L0 V1 Pn1 | G2 | MSS | LSCC |
| 5 | pT1 N0 (0/28) | G2 | MSS | RSCC |
| 6 | pT3 N0 (0/25) M1 (PER) L1 V0 Pn0 | G3 | MSS | RSCC |
| 7 | pT2 N1b (3/88) L0 V0 | G2 | MSS | RSCC |
| 8 | pT2 N0 (0/12) L1 V0 Pn0 | G3 | MSS | RSCC |
| 9 | pT3 N0 (0/15) | G2 | MSS | LSCC |
| 10 | pT3 N0 (0/19) L0 V0 Pn0 | G2 | MSS | RSCC |
| 11 | pT2 N0 (0/12) L0 V0 Pn0 | G2 | MSS | LSCC |
| 12 | pT3 N0 (0/21) L0 V0 | - | MSI | RSCC |
| 13 | pT1 N0 (0/7) L0 V0 | - | MSI | RSCC |
| 14 | pT3 N2b (8/25) M1a (HEP) L1 V1 Pn1 | G2 | MSS | LSCC |
| 15 | pT3 N0 (0/16) L0 V0 | G2 | MSS | LSCC |
| 16 | pT2 N0 (0/19) MX L0 V0 Pn0 | G2 | MSS | LSCC |
| 17 | pT3 N1a (1/17) L0 V0 Pn0 | G2 | MSS | LSCC |

**Supplementary Figure 1**


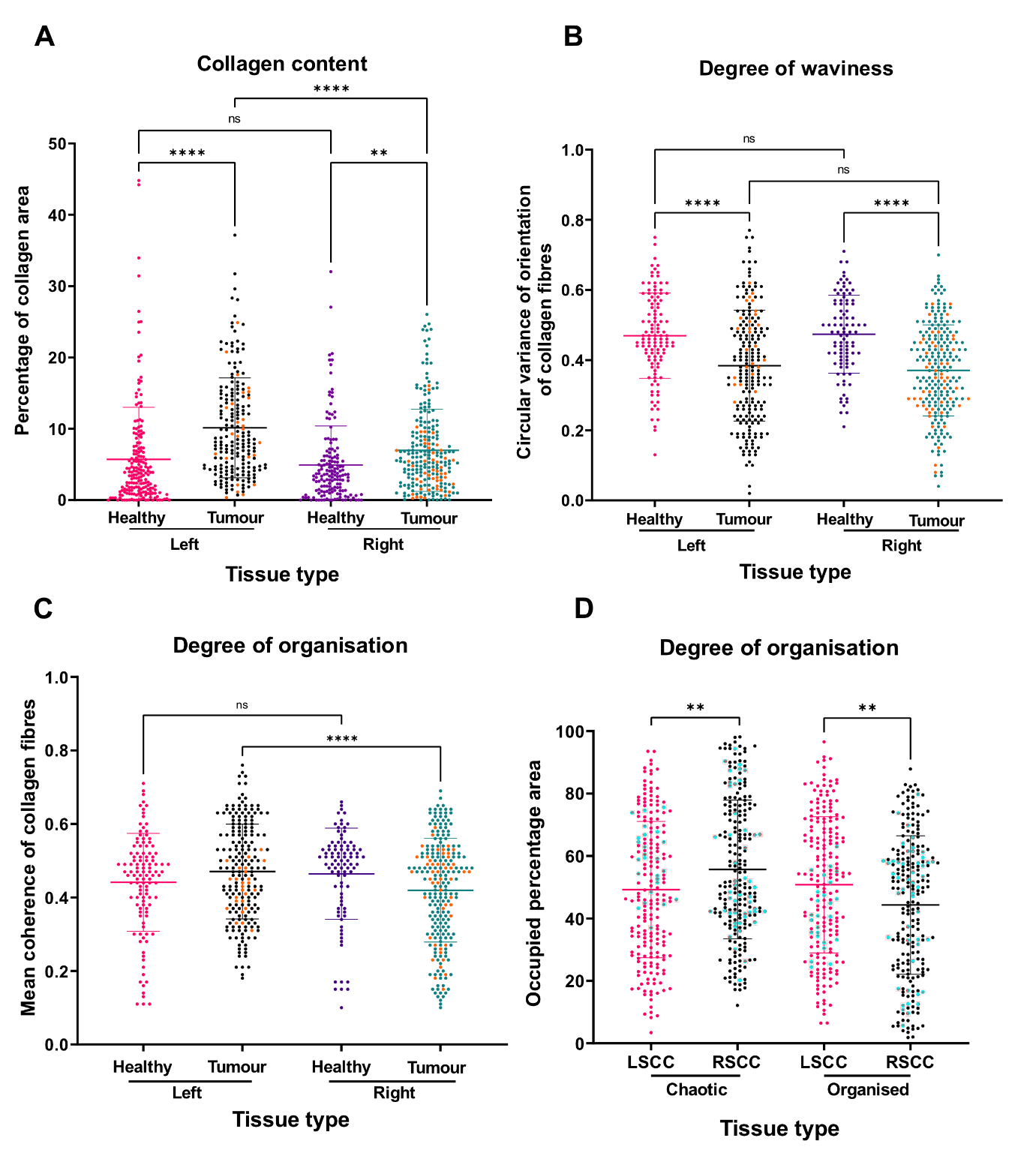


**Suppl. Figure 1: Differences in the collagen area and imaging features of collagen fibres obtained by texture analysis on the F-SHG signal from 2PLSM images.** A) Amount of collagen fibres in LSCC (n= 8) is higher than in RSCC (n=9). B) The waviness of the fibres does not change in left and right sided colon cancer. C) Coherence can distinguish the LSCC, which has a higher degree of organisation than the RSCC. D) The low coherence (chaotic) regions in RSCC are higher than LSCC. Orange (A-C) and turquois (D) points indicate ROIs from G3 tumour samples. Data is presented as mean ± SD, ** indicates p ≤ 0.01, **** indicates p ≤ 0.0001; ns = not significant.
